# Cord pilot trial - immediate versus deferred cord clamping for very preterm birth (before 32 weeks gestation): study protocol for a randomized controlled trial

**DOI:** 10.1186/1745-6215-15-258

**Published:** 2014-06-30

**Authors:** Angela Pushpa-Rajah, Lucy Bradshaw, Jon Dorling, Gill Gyte, Eleanor J Mitchell, Jim Thornton, Lelia Duley

**Affiliations:** 1 Nottingham Clinical Trials Unit (NCTU), Nottingham Health Science Partners, C Floor, South Block, Queens Medical Centre, Derby Road, Nottingham NG7 2UH, UK; 2 Neonatal Unit, Queens Medical Centre, Derby Road, Nottingham NG7 2UH, UK; 3 National Childbirth Trust, Alexandra House, Oldham Terrace, Acton, London W3 6NH, UK; 4 Maternity, Nottingham City Hospital, Hucknall Road, Nottingham NG5 1 PB, UK

**Keywords:** pilot, randomised trial, preterm birth, umbilical cord clamping, neonatal care at the bedside

## Abstract

**Background:**

Preterm birth is the most important single determinant of adverse outcome in the United Kingdom; one in every 70 babies (1.4%) is born before 32 weeks (very preterm), yet these births account for over half of infant deaths.

Deferring cord clamping allows blood flow between baby and placenta to continue for a short time. This often leads to increased neonatal blood volume at birth and may allow longer for transition to the neonatal circulation. Optimal timing for clamping the cord remains uncertain, however. The Cochrane Review suggests that deferring umbilical cord clamping for preterm births may improve outcome, but larger studies reporting substantive outcomes and with long-term follow-up are needed. Studies of the physiology of placental transfusion suggest that flow in the umbilical cord at very preterm birth may continue for several minutes. This pilot trial aims to assess the feasibility of conducting a large randomised trial comparing immediate and deferred cord clamping in the UK.

**Methods/Design:**

Women are eligible for the trial if they are expected to have a live birth before 32 weeks gestation. Exclusion criteria are known monochorionic twins or clinical evidence of twin-twin transfusion syndrome, triplet or higher order multiple pregnancy, and known major congenital malformation. The interventions will be cord clamping within 20 seconds compared with cord clamping after at least two minutes. For births with cord clamping after at least two minutes, initial neonatal care is at the bedside. For the pilot trial, outcomes include measures of recruitment, compliance with the intervention, retention of participants and data quality for the clinical outcomes.

Information about the trial is available to women during their antenatal care. Women considered likely to have a very preterm birth are approached for informed consent. Randomisation is close to the time of birth. Follow-up for the women is for one year, and for the children to two years of age (corrected for gestation at birth). The target sample size is 100 to 110 mother-infant pairs recruited over 12 months at eight sites.

**Trial registration:**

ISRCTN21456601, registered on 28 February 2013.

## Background

In the UK, infant mortality (deaths in the first year of life) for babies born very preterm (before 32 weeks gestation) is 144/1000 live births, compared to 1.8/1000 for those born at term (38 to 41 weeks) [1]. Very preterm birth accounts for 1.4% of live births in the UK, but 51% of infant deaths [1]. For infants born before 28 weeks, duration of hospital stay is 85 times longer than for term births, and hospital inpatient costs are £15,000 higher; for those born at 28 to 31 weeks, it is 16 times longer and £12,000, respectively [2].

Morbidity amongst children born very preterm is also high compared to those born at term. Of very preterm infants who survive, 5 to 10% develop cerebral palsy, and those without severe disability have a twofold or greater increased risk for developmental, cognitive, and behavioural difficulties [3,4]. Of babies born before 28 weeks, a quarter of survivors have neurosensory disability, such as cerebral palsy, severe developmental delay, blindness or deafness [5]. Surviving children have higher levels of dysfunction in a range of measures of cognition, behaviour and functioning [5], impairments that persist into adolescence and early adulthood [6,7]. Teenagers and young adults born very preterm also report poorer physical abilities and more chronic ill health and functional limitation than their peers born at term, although self-reporting of health-related quality of life is similar [8,9]. Prematurity and its sequelae have an enormous negative psychosocial and emotional impact on parents and families [5,10].

### Placental transfusion

Placental transfusion is the transfer of blood between the placenta and the baby at birth. For term births, this blood flow is usually complete by two minutes, but may continue for up to five minutes. The mean volume of placental transfusion for term births is 100 ml, which is around 29 ml/kg birth weight and 36% of neonatal blood volume at birth [11]. For preterm births, placental transfusion may take longer [12], and may be incomplete if the cord is clamped within 30 to 90 seconds [13]. This seems logical, as at term, two-thirds of the feto-placental circulation is in the infant, whilst below 30 weeks gestation, a greater proportion is in the placenta [14]. Also, the umbilical vein is smaller than at term, and uterine contraction less efficient. Cord clamping before placental transfusion is complete may restrict neonatal blood volume and red cell mass, and interrupt transition from the fetal to neonatal circulation.

As the baby is born, umbilical circulation slows and pulmonary vascular resistance falls, rapidly increasing pulmonary blood flow. This is the beginning of transition from the fetal to the neonatal circulation. For infants born too early, the mechanisms for these circulatory changes may not be fully developed and so may take longer. Continued flow in the umbilical vein and arteries at birth may be part of the physiological mechanisms assisting the baby as it makes the transition from fetal to neonatal circulation. Restricting this flow by immediate cord clamping may limit the baby’s ability to deal with this transition. If there is insufficient circulating blood volume to fill the expanding pulmonary vasculature, an infant may compensate by reducing flow to the peripheral circulation and/or to organs such as the kidney. Whilst most healthy babies at term may adapt without major consequences, for those born preterm or with their cardio-respiratory circulation already impaired, there may be substantive consequences.

Over 20 years ago it was first suggested that restricting placental transfusion by immediate clamping for preterm babies might increase the risk of intraventricular haemorrhage [15]. Possible mechanisms for this increase were suggested to be hypovolaemia, and/or increased fluctuation in blood pressure following the abrupt transition to a neonatal circulation.

Cord milking (pinching the cord close to the mother and running the fingers towards the baby, usually several times) has been suggested for preterm births as a means to increase neonatal blood volume without deferring cord clamping [16]. Cord milking over-rides the infant's physiological control of its own blood volume and blood pressure, however, and disrupts umbilical blood flow.

### Systematic review: immediate versus deferred cord clamping

The Cochrane Review of timing of cord clamping for preterm births [17] includes 15 trials, with 738 infants recruited between 24 and 36 weeks gestation. Immediate cord clamping ranged from 5 to 20 seconds, although several studies did not state the duration. Deferred clamping ranged from 31 to 120 seconds for births before 34 weeks; one study recruiting between 34 weeks and 36 weeks gestation used 180 seconds. The few studies that reported a rationale for how long to defer clamping said it had been a balance between allowing placental transfusion, and what was perceived as an acceptable delay in neonatal care. One small trial (40 mother-infant pairs) compared cord milking with immediate cord clamping [16]. Many outcomes are reported by only a few studies, so there is potential for reporting bias.

No trials reported outcome for the women. Primary outcomes for the infants were death, death or neurodisability at two years of age, ultrasound diagnosis of grade 3 or 4 intraventricular haemorrhage, and periventricular leukomalacia. Death before discharge from hospital was reported in 13 trials, and there was no clear difference between the groups (Table 1). No studies have reported death or neurodisability at two years of age. There was no clear difference between the groups in either severe intraventricular haemorrhage or periventricular leukomalacia.

**Table 1:** Immediate versus deferred cord clamping for preterm births: effects for infants

**Outcome**	**Number of trials**	**Number of participants**	**Risk ratio**	**95%confidence interval**
Death	13	668	0.63	0.31 to 1.28
intraventricular haemorrhage				
any (grade 1 to 4))	10	539	0.59	0.41 to 0.85
severe (grade 3 or 4)	6	305	0.68	0.23 to 1.96
periventricular leukomalacia	2	71	1.02	-0.52 to 5.56
temperature on SCBU admission (°C)^a^	3	143	0.14^a^	-0.03 to 0.31^a^
Transfusion				
for anaemia	7	392	0.61	0.46 to 0.81
for hypotension	4	130	0.52	0.24 to 1.11
number of transfusions^a^	5	210	-1.26^a^	-1.87 to −0.64^a^
mean arterial pressure^a^				
at birth	2	97	3.52^a^	0.60 to 6.45^a^
at 4 hours	2	111	2.49^a^	0.26 to 4.72^a^
inotropes for low blood pressure	4	158	0.42	0.23 to 0.77
necrotising enterocolitis	5	241	0.62	0.43 to 0.90
serum bilirubin peak^a^	7	320	15.01^a^	5.62 to 24.40^a^
jaundice requiring phototherapy	3	180	1.21	0.94 to 1.55
oxygen supplementation at 36 weeks	5	209	0.69	0.42 to 1.13

^a^mean difference.

SCBU, special care baby unit.

Infants allocated to deferred cord clamping had fewer diagnoses of any intraventricular haemorrhage than those allocated to immediate clamping (Table 1). There was no clear difference between the groups in the three trials reporting temperature on admission to the special care baby unit. Deferred cord clamping was associated with less transfusion for anaemia, but there was no clear difference in transfusion for hypotension. Deferred clamping was also associated with higher mean arterial blood pressures at birth and at four hours of age, and less requirement for inotropes. It was also associated with a reduction in the risk of necrotising enterocolitis. Infants allocated deferred cord clamping had higher serum bilirubin, but there was no clear difference in jaundice requiring phototherapy in the three trials reporting this outcome. There was no clear difference between the groups in oxygen requirement at 36 weeks postmenstrual age.

Follow-up at age seven months (corrected for gestation at birth) was reported for one study, which recruited 72 infants. Of these, five died before seven months and nine were lost to follow-up. There was no overall difference between the groups in Bayley Scales of Infant Development-II.

The review concludes that ‘to reliably compare strategies for influencing placental transfusion we need large high-quality trials, with sufficient power to reliably assess clinically relevant differences in important outcomes’ [17].

### Initial care at birth for very preterm infants: at the bedside or at the room side

Initial neonatal care and stabilisation takes place on a resuscitaire. Traditionally, this is at the side of the room, or in an adjacent room. Disadvantages of these locations are that they necessitate immediate cord clamping, and that often the woman and her partner are not able to see or touch their baby at birth [18,19]. If cord clamping is deferred this should not necessarily mean that neonatal care is also deferred. Strategies for providing neonatal care and stabilisation of the baby at the bedside have now been developed. Parents’ views following initial care at the bedside have been positive [20]. Evaluation of the views of clinicians is also positive, although there are initial issues around training, practical arrangements for preparing and moving the equipment, and making space at the bedside [20]. The experience of providing initial neonatal care at the bedside has been positive, and it is now part of standard care in some hospitals.

Providing neonatal care for very premature infants at the bedside allows the woman and her partner to share the first moments of their child’s life [21,22], if they wish to, and is therefore potentially a more family-centred approach. Family-centred care in neonatal units, with improved communication and involvement of parents in their baby’s care, appears to benefit babies, is welcomed by parents [10] and is an NHS priority [23]. Providing neonatal care at the bedside has parallels with family presence during resuscitation of adults and children, which is preferred by families and appears to be beneficial [24-28].

### Current practice for timing of cord clamping at very preterm births

In the UK, 57% of obstetricians report clamping the cord within 20 seconds for very preterm births [29]. Just 15% of midwives and 5% of obstetricians reported that they routinely record when the cord was clamped in the medical notes. Guidelines for care during the third stage of labour make various recommendations, and it is often not clear how these should be applied to very preterm babies, many of whom will require neonatal care at birth [30-32].

### Why a trial is needed now

Current evidence is that for very preterm births, timing of cord clamping, and other strategies to influence placental transfusion, may improve outcome at hospital. But the trials are small, and overall there is high risk of bias. The effects on substantive outcomes and long-term neurodevelopment remain uncertain. Assessing alternative strategies for timing of cord clamping has been identified as a research priority by service users [33], researchers [17,34,35], obstetricians [29], midwives [29], neonatologists (Duley L, Farrar D, McGuire W, Oddie S: Survey of the Extended Neonatal Network to assess views on timing of cord clamping and placental transfusion, unpublished), NICE [30,36], and the Royal College of Obstetricians and Gynaecologists [21].

Our primary hypothesis is that for children born before 32 weeks gestation immediate cord clamping is associated with higher death or neurosensory disability at two years of age (corrected for gestation at birth) than deferred cord clamping. A trial to test this hypothesis would need to be large and multicentre. This protocol is for a pilot trial to assess the feasibility such a study.

## Methods/Design

This study is a pragmatic multicentre pilot randomised trial comparing alternative strategies for cord clamping at very preterm birth.

### Participants

Women are eligible for the study if they are expected to have a live birth before 32 weeks gestation, regardless of mode of birth or whether cephalic or breech presentation.

Exclusion criteria are monochorionic twins (from an ultrasound scan) or clinical evidence of twin-twin transfusion syndrome, triplets or higher order multiple pregnancy, or known major congenital malformation.

### Interventions

There is no consensus about the definition of immediate or deferred cord clamping, or about the optimal timing of cord clamping for very preterm birth. We have chosen our interventions based on current practice [29], the interventions reported in the trials included in the Cochrane review [17], consultation with neonatologists, and our work measuring the volume and duration of placental transfusion [11,37].

The interventions are cord clamping within 20 seconds or cord clamping after at least two minutes.

For both groups, while the cord is intact, the baby should be at the level of the placenta, and should not be lifted above the level of the mothers’ abdomen. All other aspects of care are at the discretion of the attending clinicians, including administration of a prophylactic uterotonic drug.

For deferred cord clamping, care for the baby is provided at the mother’s bedside. For immediate clamping care is either at the bedside or at the side of the room, at the discretion of the attending clinicians. In both cases the baby receives the same care at birth, just in different places. Neonatal care is based on local unit policy and consistent with newborn life support guidelines [32,38]. Standard equipment is used for both groups according to local practice, including plastic sheets or bags (depending on gestation and local practice), towels and any other equipment such as hats, warming mattress or overhead heaters, and saturation monitors.

For neonatal care at the bedside, babies are placed onto a firm surface next to the mother’s bed or to the operating theatre table, with easy access to necessary equipment. This is achieved either by moving the conventional resuscitaire alongside the woman’s bed [39] or by using a small specialised mobile trolley (for example the BASICS trolley) [40].

### Outcome measures

To assess the feasibility of a large multicentre trial, the outcomes for this pilot trial are as follows:

1. number of women recruited in each hospital;

2. proportion of potentially eligible women recruited;

3. reasons for non-recruitment (medical, parental, logistic, other);

4. spectrum of gestational age and neonatal outcome among recruits;

5. compliance with the trial interventions, and reasons for non-compliance;

6. completeness of data collection for main outcomes;

7. views of women and their partners on recruitment, randomisation and the interventions’ and

8. proportion lost to follow.

Clinical outcomes for infants and women likely to be used in the main trial will also be collected. These are death or neurosensory disability at age two years (corrected for gestation at birth) as primary outcome. Secondary outcomes for the baby are death, blood transfusion, intraventricular haemorrhage (grade 3 to 4), periventricular leukomalacia, hypothermia, respiratory distress syndrome, ventilation, necrotizing enterocolitis, treatment for hyperbilirubinemia, duration of hospital stay and neurosensory delay at age 2 years (corrected for gestation at birth).

Secondary outcomes for the women are postpartum haemorrhage, infection, depression, and breast feeding/expressing.

Outcome for the women is assessed at discharge from hospital, by a self-completed (either handed out in hospital or sent by post) questionnaire at four to eight weeks, and by self-completed postal questionnaire at one year. For the babies, outcome is assessed at 36 weeks postmenstrual age, at discharge from hospital, and at age 2 years (corrected for gestation at birth). This assessment at age two includes an Ages and Stages Questionnaire [41], the PARCA-R (Parent Report of Children’s Abilities-revised) questionnaire [42], and a Bayley Scales of Infant Development III [43]. This Bayley assessment is blind to the allocated group.

### Sample size

Eight large maternity hospitals are included in this pilot trial, with an estimated total of 43,600 live births per year (5 to 6,000 average annual live births per unit). In the UK, 1.4% of live births are before 32 weeks gestation [1]; we, therefore, expect 610 potentially eligible births and anticipate recruitment of between 100 and 110 women (16 to 18% accrual) over 12 months.

### Randomisation

Randomisation is in a 1:1 ratio, stratified by hospital. Sequence generation uses computer-generated, random permutated balanced blocks of randomly varying size, created by the Nottingham Clinical Trials Unit (NCTU) in accordance with their standard operating procedure. Concealment of allocation is by sealed consecutively numbered opaque envelopes. These envelopes are held in a ring binder randomisation folder, which is kept in a secure place that is easily accessible for the delivery suite. Envelopes are removed in consecutive order by tearing the next one out of the randomisation folder at the punch holes. At the time of removing the folder, whoever takes it initials and dates the folder log. On the front of each envelope is a reminder to check eligibility, and a label to be completed *before* the envelope is opened. This label records the date, time, woman’s initials, her date of birth, and her gestation. Once this information is complete, the woman is considered to be randomised, even if the envelope is not opened.

The envelopes contain a yellow card stating when the cord should be clamped; stickers for the woman and baby’s medical notes (indicating they are in the trial); and a Birth Record (plus a second to be used for twin births) to be completed at the time of birth and filed in the baby’s medical notes. The Birth Record asks for information about care during the third stage of labour and initial neonatal care. Each delivery suite has a Cord Pilot Trial secure mailbox. After each randomisation, the randomisation envelope and the yellow allocation card are posted into this mailbox. The mailbox is checked daily by the research midwife/nurse. Details from the randomisation envelope are then entered into the online randomisation log maintained by the NCTU, and the envelope is filed with the woman’s Case Report Form.

If an envelope is taken from the randomisation folder but not used, the discarded envelope is posted unopened into the Cord Pilot Trial mailbox. These unused and discarded envelopes are notified to NCTU, and the envelopes returned to NCTU. All randomisation envelopes, both opened and unopened, are accounted for by the NCTU. Envelopes used out of order or tampered with will be recorded and reported.

### Trial entry and recruitment

General information about the trial, such as summary information leaflets and study posters, is available on antenatal clinics and antenatal wards. These introduce the trial and explain how women can access more information if they wish. To avoid causing unnecessary anxiety this information makes clear that only around 1 in 70 women give birth before 32 weeks gestation. Women who would like more information are offered the parent information sheet, which is more detailed.

**Figure 1:**
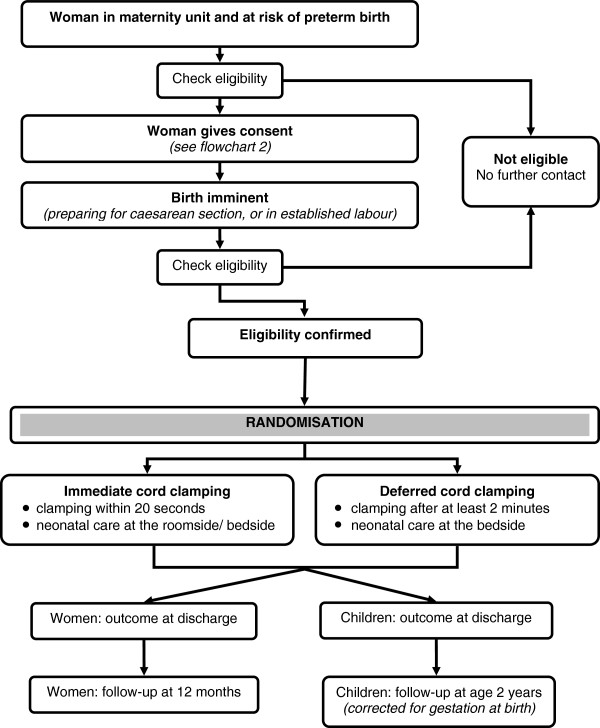
**Participant flow.** This figure shows the participants’ pathway through the trial.

**Figure 2:**
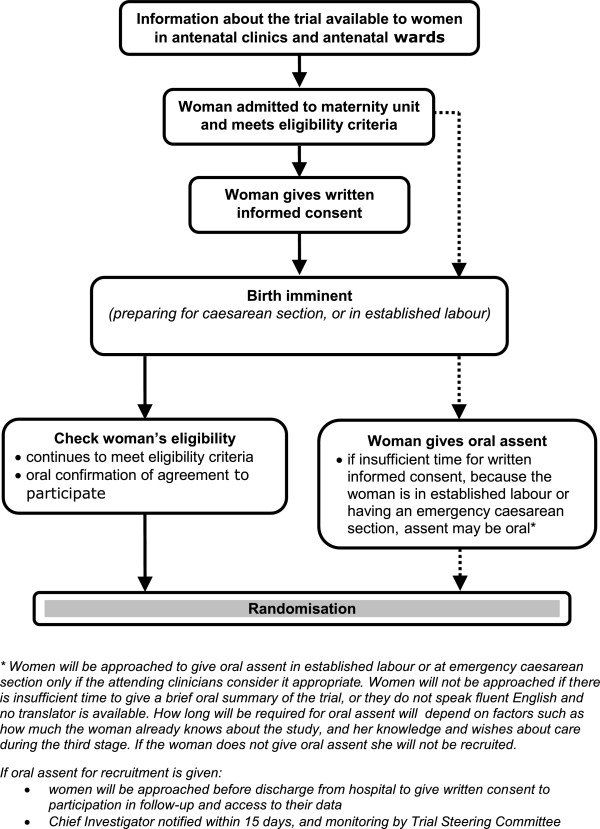
**Consent pathways.** This figure shows the two different consent pathways used within the trial.

Women in the antenatal clinics, antenatal wards or a high risk antenatal clinic, who are at a high risk of having a birth at less than 32 weeks gestation are offered the parent information sheet. They have an opportunity to discuss the study with their family and partner. If a woman decides to participate in the trial, written informed consent is taken. Whenever possible, at least 12 hours is given for the woman to consider participating in the trial. If a woman does not participate, this decision will not influence her clinical care.If a woman who has consented to participate in the trial is in established labour, or is being prepared for a caesarean section, her gestation and willingness to participate are checked. If she still meets the inclusion criteria she is randomised (Figure 1). Randomisation near to the time of birth should ensure that women enrolled into the trial give birth before 32 weeks gestation. However, if a woman is randomised but does not give birth until after 32 weeks gestation, she remains in the trial.If there is insufficient time to gain written consent before randomisation, and the attending clinician feels it is appropriate, these women will be asked verbally if they would like to participate in the trial. After a brief summary of the study as well an opportunity to ask questions, the woman is asked if she would like to participate in the trial. If she agrees, she is then randomised (Figure 2). This is recorded in her medical notes, and the clinician taking consent completes a form. Before discharge from hospital, the woman is approached for written consent to use her and her baby’s data, and for participation in follow-up. If a woman declines oral assent, she is not randomised.

This process for oral assent was developed in discussion with the National Childbirth Trust (NCT) and Bliss, the special care baby charity. It is also in line with recommendations on valid consent for research while in labour from the Royal College of Obstetricians and Gynaecologists [44].

### Follow-up of participants

At discharge from hospital, outcome data for the women and babies are collected from the medical notes, along with contact details to facilitate follow-up. Women are asked to complete a questionnaire at four to eight weeks. If the baby is still in hospital this is given to them by the research midwife/nurse, or if the baby is no longer in hospital, it is posted to their home address. The questionnaire includes the Hospital Anxiety and Depression Scale (HADS) [45], questions asking the women about their and her babies’ care and their experience in hospital, and asks women about their experience of participating in the CORD Pilot trial. There is also a section asking women about their baby’s feeding (removed if the baby died). When the child is one year old, a birthday card is sent along with a second questionnaire asking the same questions. A stamped addressed envelope is provided to return questionnaires.

When the baby is 2 years old (corrected for gestation at birth), the parents/carers are contacted to arrange a neurodevelopment assessment for the child, using the Bayley Scales of Infant Development III. Four weeks before this assessment, the family are sent the Ages and Stages Questionnaire by post [41], and asked to complete and return this before the assessment (using a prepaid envelope). The assessment is by a trained practitioner either at home or in a clinic, whichever is preferred by the family. During the visit, the parents are asked to complete the Parent Report of Children’s Abilities - Revised (PARCA-R) [42].

For all questionnaires, if there is no response a reminder is sent after two weeks. If there is still no response after another two weeks, the coordinating centre telephone to offer the opportunity to complete the questionnaire over the telephone. If no telephone number is available another postal reminder is sent. Stamped addressed envelopes are provided for all questionnaires.

Women and babies are also ‘flagged’ with the Health and Social Care Information Centre, through the Medical Research Information Service. This allows identification of deaths after discharge from hospital and movement out of the NHS, information that helps prevent inappropriate contact with the family and also reduces losses to follow-up.

### Adverse events

This study includes babies who are high risk for adverse outcomes, and so adverse events that could be influenced by the trial interventions are being collected as outcomes for the study.

Although neonatal or infant death is one of the outcomes for this study, death before discharge from hospital is considered as a serious adverse event (SAE). Any unexpected and serious adverse event, for either the woman or the baby, considered to be potentially related to the study interventions will also be reported as a SAE. Any SAE that is not a death will be followed until there is resolution or the event is considered stable.

All SAEs are reported to the Chief Investigator. The Chief Investigator will submit, every six months for the duration of recruitment to the trial or on request, a safety report to the Data Monitoring Committee, which will include all reported SAEs.

### Site set-up and training

Site set-up and training includes a range of strategies, such as collaborators meetings, site initiation visits, video and simulations, slide sets, and site monitoring visits. Training in providing neonatal care at the bedside is provided for all clinical staff, including neonatologists, obstetricians, and midwives.

### Feasibility criteria for progression to the main trial

The final decision about progression to the main trial will be made by the independent Trial Steering Committee, in consultation with the Data Monitoring Committee. The pre-specified criteria for feasibility are based on:

1. recruitment of at least 50% of target at 12 months. But, if recruitment is below 80% of target, there will need to be clear and achievable strategies for overcoming identified barriers thereby improving recruitment.

2. at least 80% of women in each group receiving the intervention to which they had been allocated during the final six months of recruitment (as compliance may be lower in the early stages of a feasibility study). For a proportion of births it is anticipated that the cord will be too short to allow initial care at the bedside, and so for these infants in the deferred arm cord clamping may be before 2 minutes. For example, in a study requiring babies to be placed on weighing scales with the cord intact, of 33 term births for 5 (15%) the cord was too short to allow the baby to be placed on the scales [11]. For the deferred arm, feasibility criteria will be considered to be met if 80% of infants with adequate cord length had cord clamping after at least 2 minutes.

3. the difference between the two groups in median time to cord clamping is at least 45 seconds.

### Data management and trial monitoring

Data are entered as they accumulate into a trial specific database developed and maintained by the NCTU. Access to the database will be restricted and secure. All trial data are anonymised by use of unique participant trial numbers. Data quality is checked using criteria for out-of-range and consistency, and checks for conflicting data within and between data collections forms. Missing data and data queries are referred promptly back to the recruiting site for clarification.

For the follow-up phase, identifiable information about participants will be held in a separate database to the trial data. Access to this information will be restricted to those involved in the follow up phase, as authorised by the Chief Investigator.

Trial monitoring is by central statistical monitoring combined with site visits. Central statistical monitoring is used to monitor patterns of recruitment at sites, reasons for non-recruitment of potentially eligible women, characteristics of women recruited, gestation at recruitment, and time of recruitment. It is also to assess compliance with the protocol, such as eligibility and compliance with the trial interventions. Each site has at least one monitoring visit; timing of this visit is influenced by recruitment, data quality, and compliance with the protocol and study procedures.

The Chief Investigator is the custodian of the data. The trial is conducted in compliance with the current revision of the Declaration of Helsinki (last amendment October 2008), with relevant regulations, and with MRC Guidelines for Good Clinical Practice in Clinical Trials, [46] which is based on International Conference on Harmonisation (ICH) guidelines for good clinical practice (GCP) (CPMP/ICH/135/95) July 1996.

### Data analysis

Eligibility, recruitment and retention through the study will be presented in a CONSORT flow diagram to show the number of eligible women, the number approached for participation (split according to written consent and oral assent), the number of women (and their babies) randomised into the two groups, and completion of follow-up of the outcomes for the main study. Reasons for eligible women not providing consent and for those who have given consent not being randomised will be described, when known. The time between oral assent and randomisation will be analysed to check that this method of consent is being used appropriately. Reasons for loss to follow-up after discharge from hospital will also be presented, where known.

Baseline characteristics of the women (age, previous pregnancy history, reason for preterm birth) and the baby at birth (gestational age at birth, baby gender and birth weight) will be presented descriptively split by allocated group. Continuous data will be summarised in terms of the mean, standard deviation and/or median, lower and upper quartiles, minimum and maximum with the number of observations. Categorical data will be summarised in terms of frequency counts and percentages. The number of missing values will be presented where applicable.

Compliance with the intervention will be assessed by summarising the median time to cord clamping in the two groups, the difference in median time to cord clamping between the two groups, and the proportion of women receiving the allocated intervention. Reasons for non-compliance with the allocated intervention will be tabulated. Compliance will be assessed for the first six months of recruitment and for the second six months, as our hypothesis is that compliance will improve over time. The number (and percentage) of babies receiving bedside or room side care and the type of care given in each location in the two groups will be described.

Outcomes will be described for women at discharge, at four to eight weeks, at 1 year, and for babies at discharge and at 2 years. Any unexpected serious adverse events for either the women or the baby will also be described. This description of outcome will be for both groups combined, not by allocated group. If feasibility is demonstrated, and the trial progresses to a main study outcome by allocated group will remain confidential to the Data Monitoring Committee. It would then contribute to the sample size for the main trial.

The woman’s views about whether participation in the Cord Pilot Trial will be tabulated overall, and split by characteristics that may influence the women’s views about participating in the study. These characteristics are pre-specified as allocated group, death of the baby, whether oral assent was used, maternal age at recruitment, gestation at recruitment less than 30 weeks, severe post-partum haemorrhage, postnatal depression, length of stay in a special care baby unit longer than six weeks and whether participants needed a reminder to complete the questionnaires.

### Archiving

Data and all appropriate study documentation will be stored for a minimum of 10 years after completion of the trial, including the follow-up period. The trial master file and trial documents held by the Chief Investigator on behalf of the sponsor will be archived in secure archive facilities at Nottingham University Hospitals NHS Trust. This archive will include all trial databases and associated meta-data encryption codes.

### Trial management

Day-to-day management of the trial is the responsibility of the Trial Management Group (TMG), which meets at least every two months and more often if required. Trial oversight is by an independent Trial Steering Committee. Safety of trial participants is monitored an independent Data Monitoring Committee, who report to the Trial Steering Committee. Trial coordination is through the NCTU.

### Ethics approval

Approval for this study was granted by the Nottingham 2 Research Ethics Committee (NRES reference 12/EM/0283).

### Sponsor

Nottingham University Hospitals NHS Trust acts as the main sponsor for this trial. Delegated responsibilities are assigned to the NHS trusts taking part. Standard NHS Indemnity applies.

## Discussion

Recruitment to the Cord Pilot Trial began in March 2013 and will close on 28 February 2014. Assessment of the feasibility outcomes when recruitment is complete will inform the decision about progressing to a large multicentre trial in the UK.

## Trial status

Recruitment to the Cord Pilot Trial is ongoing. The feasibility assessment is based on recruitment from 1 March 2013 to 28 February 2014. Recruitment is being continued in pilot sites whilst main trial funding is sought.

## Abbreviations

ASQ: Ages and Stages Questionnaire; HADS: Hospital Anxiety and Depression Scale; ICH GCP: International Conference on Harmonisation for good clinical practice; MRC: Medical Research Council; NCT: National Childbirth Trust; NCTU: Nottingham Clinical Trials Unit; NIHR: National Institute of Health Research; NRES: National Research Ethics Service; PARCA-R: Parent Report of Children’s Abilities-revised; RCOG: Royal College of Obstetricians and Gynaecologists; SAE: serious adverse event; TMG: Trial Management Group.

## Competing interests

The authors declare that they have no competing interests.

## Authors’ contributions

LD conceived the study and is the chief investigator and project lead. JD, JT, GG made substantial contributions to the conception and design of the work, revised this work critically for important intellectual content and approved the final version to be published. LB will provide statistical support to the study and conduct the data analysis. LD and LB drafted this work, revised it critically for important intellectual content and approved the final version to be published. AP is the Trial Manager and EJM is the Senior Trial Manager. Both AP and EJM will be responsible for the acquisition and interpretation of data for the trial, and both have drafted this work and approved the final version to be published. LD, GG and JT are grant holders, and JD is a collaborator on the grant application. All authors read and approved the final manuscript.
